# Bridging the gaps in fibroblast activation protein targeted radionuclide therapy: a translational perspective

**DOI:** 10.1007/s00259-025-07711-3

**Published:** 2026-02-02

**Authors:** Chunhui Wu, Ahmad Kurniawan, Zihe Ming, Ines F. Antunes, Walter Noordzij, Andor Glaudemans, Bart Cornelissen

**Affiliations:** Department of Nuclear Medicine and Molecular Imaging, University of Groningen, University Medical Center Groningen, Groningen, the Netherlands

**Keywords:** Fibroblast activation protein (FAP), Targeted radionuclide therapy (TRT)

## Abstract

Fibroblast activation protein-targeted radionuclide therapy (FAP-TRT) has emerged as a novel strategy for modulating the tumour microenvironment (TME) by selectively eradicating FAP-expressing cancer-associated fibroblasts (CAFs). Although preclinical studies have demonstrated promising results across various tumour models using diverse radiolabelled FAP inhibitors, clinical translation remains limited by modest efficacy, short tumour retention, and highly heterogeneous responses. This review aims to provide an overview of recent advances in radiopharmaceutical design to enhance tumour targeting and prolong retention. Furthermore, we summarise early clinical findings and ongoing trials, while emphasising the potential translational challenges of FAP-TRT. Special emphasis is placed on the radiobiological underpinnings of FAP-TRT, including the impact of CAFs heterogeneity, potential pro-tumorigenic effects of sublethal irradiation, and the uncertain contribution of bystander and abscopal effects. We further highlight the need for the development of translationally relevant tumour models, optimised dosimetry, predictive biomarkers, and refined patient selection criteria. Finally, we propose future directions such as combination therapy with immune checkpoint inhibitors (ICIs). Together, these insights aim to bridge the gap between promising preclinical efficacy and limited clinical outcomes in FAP-TRT.

## Introduction

In the evolving landscape of cancer therapeutics, targeted radionuclide therapy (TRT), also referred to as radiopharmaceutical therapy, radioligand therapy, molecular radiotherapy, or internal radiation therapy, has emerged as a transformative and promising treatment strategy for managing unresectable and metastatic tumours [1, 2]. The fundamental principle of TRT relies on the targeted delivery of radiopharmaceuticals to tumour-specific molecular markers, where they emit ionising radiation to cause damage to tumour cells and the surrounding tumour microenvironment (TME). This spatially confined energy deposition induces localised tumour cell death through direct DNA damage and indirect radiobiological effects, while largely sparing adjacent healthy tissues [3, 4]. Recent clinical advances demonstrated significant progress in TRTs, particularly in the treatment of neuroendocrine tumours through somatostatin receptor-targeted approaches such as [^177^Lu]Lu-DOTATATE and prostate cancer management using prostate-specific membrane antigen (PSMA)-directed radiopharmaceuticals [5, 6]. Fibroblast activation protein (FAP), recognised as a pan-tumour marker, is overexpressed on cancer-associated fibroblasts (CAFs) within the tumour stroma, as well as on some malignant epithelial and mesenchymal tumour cells [7, 8]. Overexpression of FAP on CAFs has been shown to correlate with the proliferation, invasion, and drug resistance of malignant tumours [9, 10], which highlights its potential as a versatile target for cancer diagnosis and therapy. In recent years, FAP-based TRT has gained substantial attention in the field of oncology. Its therapeutic efficacy is believed to arise from two distinct yet complementary mechanisms: (1) direct elimination of CAFs, thereby suppressing their tumour-promoting functions in extracellular matrix remodelling, immune evasion, and treatment resistance; and (2) the crossfire effect, which enables energy deposition to reach adjacent tumour cells beyond the immediate target site [11, 12]. Despite compelling preclinical evidence demonstrating the efficacy of FAP-TRT in inhibiting tumour growth across various murine models [13–15], clinical translation has revealed its limitations. Most studies reported relatively low objective response rates (ORRs); the median ORR across all published FAP-TRT studies is only 21.4%, with most studies reporting ORRs below 30% and several showing no partial responses at all [16–36]. Only two small-scale studies (i.e., in sarcoma or lung cancer), each involving fewer than 10 patients, reported ORRs exceeding 40% [17, 21]. In contrast, [^177^Lu]Lu-DOTATATE and [^177^Lu]Lu-PSMA have demonstrated more robust clinical efficacy. For example, [^177^Lu]Lu-DOTATATE TRT in well-differentiated neuroendocrine tumours has shown ORRs typically exceeding 30%, with several studies reporting response rates ranging from 40% to 70% [37]. Similarly, in patients with metastatic castration-resistant prostate cancer (mCRPC), [^177^Lu]Lu-PSMA therapy has demonstrated a pooled ORR of about 48% in a recent meta-analysis [38]. This disconnect between robust preclinical promise and underwhelming clinical outcomes highlights a critical translational challenge unique to stromal**-**TRT. While some current studies focus on improving tumour retention by modifying ligand structures or introducing functional groups between radionuclides and targeting moieties, others aim to enhance therapeutic efficacy by exploring different types of radionuclides—including alpha, beta, and Auger emitters [13, 39]. These efforts seek to identify the optimal combination of targeting vector with radionuclide properties. However, such technical refinements alone may not be sufficient to overcome the fundamental clinical challenges. To address the disconnect between robust preclinical results and modest clinical efficacy, future research should prioritise a deeper understanding of tumour biology — particularly the heterogeneity of FAP expression, the diverse biological role of FAP-expressing CAFs and the impact of ionising radiation on their behaviour — to guide the development of more refined strategies for patients.

Against this background, this review aims to offer a comprehensive and translational perspective on FAP-TRT, integrating insights from radiobiological principles, radiopharmaceutical design strategies, and clinical research. We examine the discrepancies between encouraging preclinical outcomes and the modest efficacy observed in early clinical trials, with particular attention to the biological heterogeneity of CAFs, tumour retention challenges, and model-related limitations. Beyond preclinical optimisation, we summarise current clinical studies and ongoing trials, assess early safety and efficacy signals, and identify barriers to clinical translation such as suboptimal dosimetry, off-target toxicity, and the lack of validated biomarkers. Finally, we propose evidence-based strategies to enhance therapeutic performance—including improving tumour targeting specificity, maximising radiation delivery, and integrating immune modulation—to better bridge the gap between preclinical promise and clinical reality.

## Preclinical studies on FAP-TRT

**Fig. 1:**
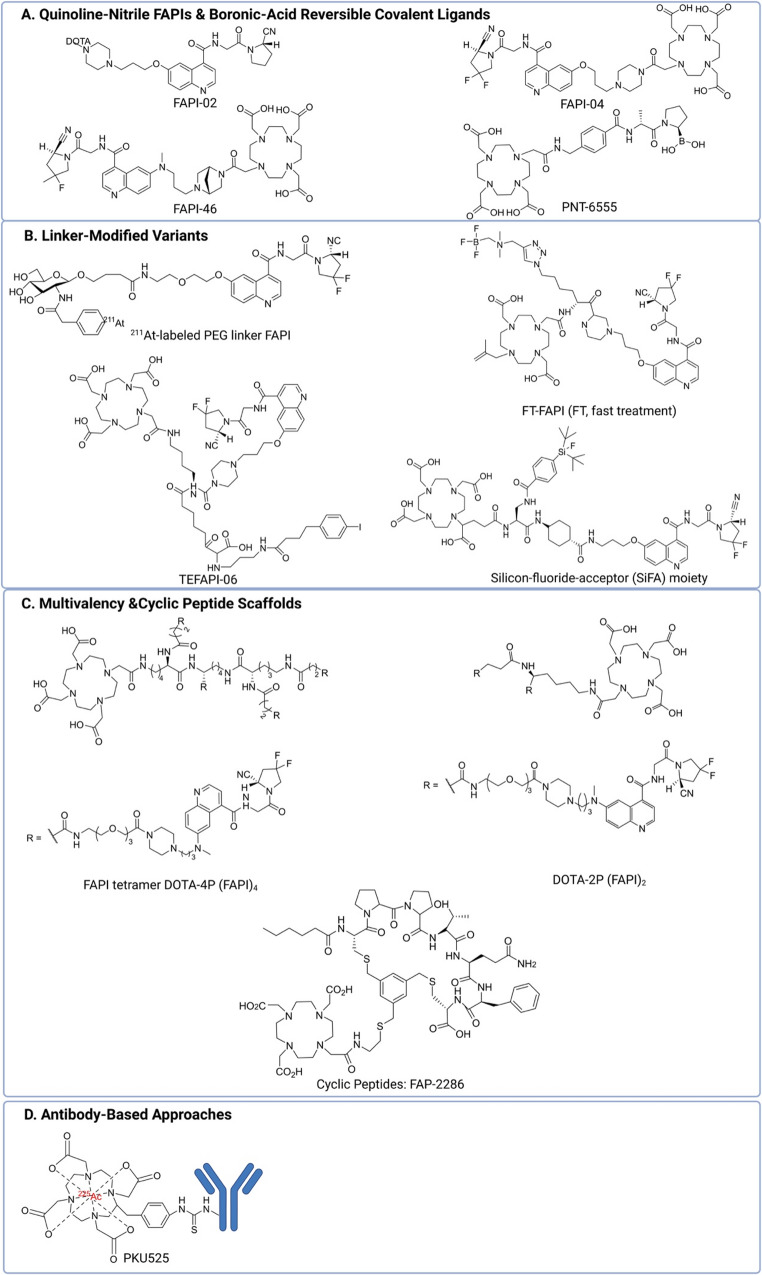
Representative FAP-targeted ligand designs for targeted radionuclide therapy

To identify the key preclinical and clinical studies discussed in this review, a non-systematic (narrative) PubMed search was conducted. Because FAP-TRT expanded rapidly in this period, Tables 1 and 2 are intentionally limited to 2020–2025; earlier foundational studies are cited narratively for context but are not tabulated. The search used combinations of keywords such as “fibroblast activation protein (FAP)”, “FAPI”, “radionuclide therapy”, “targeted radionuclide therapy (TRT)”, “radioligand therapy (RLT)”, “peptide receptor radionuclide therapy (PRRT)”, and “radiopharmaceutical”. Studies were then manually screened to include only those reporting preclinical in vivo experiments evaluating tumour uptake, retention, dosimetry, or therapeutic efficacy of FAP-targeted radionuclide therapies. Key preclinical studies are summarised in Table 1, highlighting the diversity of FAPI-based compounds, radionuclides, and tumour models used to investigate tumour retention and treatment effects. Representative designs of FAP-targeted ligands and their structural modifications are illustrated in Fig. 1. In the subsequent sections, we provide an overview of recent preclinical efforts to optimise FAP-targeted radionuclide therapy, focusing on improvements in the targeting moiety, radioligand design, and combination approaches with immune checkpoint inhibitors (ICIs).

**Table 1:** Preclinical studies of FAP-targeted radionuclide therapy in last five years (2020–2025)

Year	Author	Radiopharmaceuticals	Drug characteristics	Tumour model	Maximum injected activity (MBq)	Cycles	Tumour retention time	Absorbed dose	Response	Control group	Immune effects	Adverse event
2025	Wang et al.	[¹⁷⁷Lu]Lu-FAP-HXN	β-emitter, peptide monomer	HEK-293-FAP xenografts	29.6	Single dose	7.09 ± 0.75%ID/g 120 h p.i.	N/A	Significant tumour inhibition and better survival rate	Vehicle and group treated by [^177^Lu]Lu-FAP-2286	N/A	No toxicity
2025	Shirpour et al.	[^135^La]La-FAP-2286	Auger electrons emitter, cyclic peptide	HEK-293-FAP xenografts	7.4	Single dose	9.3% ID/g at 48 h p.i.	N/A	N/A	Vehicle and group injected with [^135^La]LaCl_3_	N/A	N/A
2025	Liu et al.	[¹⁷⁷Lu]Lu-DOTAGA.(SA.FAPi)_2_	β-emitter, dimer	4T1 syngeneic tumour model	18.5 (10 nmol/kg and 50 nmol/kg)	Single dose	N/A	N/A	Lower molar dose led to better inhibition	Vehicle, different molar doses	N/A	No toxicity
2025	Chen et al.	[¹⁷⁷Lu]DOTA-2P(FAPI)_2_	β-emitter, dimer	CT26-FAP syngeneic model	18.5	Single dose	~ 1.5% ID/g at 72 h p.i.	N/A	100% complete tumour regression (with PD-L1)	Vehicle, different combination of FAP-TRT and PD-L1 inhibit/ neutrophil blockade / CD8 + Tcell blockade	CD8 + T cells↑, Tregs↓	No toxicity
2025	Ceuppens et al.	[¹³¹I]I-GMIB-4AH29, [²²⁵Ac]Ac-DOTA-4AH29	α and β-emitter, single domain antibodies	TC-1-hFAP lung tumour xenografts	39.3 ([¹³¹I]I-GMIB-4AH29), 0.0216 ([²²⁵Ac]Ac-DOTA-4AH29)	6 injections	[¹³¹I]I-GMIB-4AH29: 0.9 ± 0.1%ID/g; [²²⁵Ac]Ac-DOTA-4AH29: 1.7 ± 0.2%ID/g at 24 h p.i.	¹³¹I: 13.3 Gy (tumour), 28.9 Gy (kidneys); ²²⁵Ac: 2.4 Gy (tumour), 10.8 Gy (kidneys)	Delayed tumour growth, increased survival (²²⁵Ac > ¹³¹I)	Vehicle	PD-L1↑, CD8 + T cells↑, LAG-3↑	²²⁵Ac caused weight loss at high doses
2024	Cui et al.	[^177^Lu]Lu-FAPI-mFS, [²²⁵Ac]Ac-FAPI-mFS	β-emitter, FAPI modified with meta-fluorosulfate SuFEx warhead	HT-1080-FAP xenograft and SDC-PDX	33.3 ([^177^Lu]Lu-FAPI-mFS), 0.0222 ([²²⁵Ac]Ac-FAPI-mFS)	2 injections	12.1 ± 1.2%ID/g at 5 h p.i.	Tumour: 890 mGy/MBq	~ 100% complete response (CR)	Vehicle and [^177^Lu]Lu-FAPI-04, [²²⁵Ac]Ac-FAPI-04	N/A	No significant toxicity
2024	Zhao et al.	[^177^Lu]Lu -LNC1004 (EB-FAPI)	β-emitter, albumin-binding	MC38/NIH3T3-FAP, CT26/NIH3T3-FAP syngeneic model	18.5	2 injections	~ 1% ID/g at 144 h p.i.	N/A	Complete response in MC38 model (100%), partial in CT26 (3/8, 37.5%), 100% rejection upon rechallenge	Vehicle, PD-L1 inhibitor, [^177^Lu]Lu-LNC1004, combination	PD-L1↑, CD8 + T cells↑, M1 macrophages↑	No long-term toxicity
2024	Ye et al.	[²¹¹At]At-APBA-FAPI	α-emitter, albumin-binding	U87MG glioma xenografts	1.11	Single dose	6.36 ± 3.73% ID/g at 6 h p.i.	N/A	Significant tumour growth inhibition (95%)	Vehicle	N/A	High dose caused thyroid toxicity and weight loss
2024	Wang et al.	[¹⁷⁷Lu]Lu-DOTA-PEG-PDA-FAPI	β-emitter, nanocarrier-based	U87MG glioma xenografts	12.95 (intratumoural injection and intravenous injection, respectively)	Single dose	182.6 ± 73.8%ID/g 5 days (intratumoural), low uptake (i.v.)	N/A	Significant tumour growth inhibition (i.t.), no response (i.v.)	Vehicle	N/A	No toxicity
2024	Taddio et al.	[²²⁵Ac]Ac-FAPI-46	α-emitter, small molecule	FAP FSA fibrosarcoma syngeneic model & corresponding immunosuppression model	0.060	3 injections	Short retention with only 20% of the maximal tumour activity remaining after 24 h)	N/A	Combined with PD-1 ICB: 18% partial tumour regression, 54.5% delayed tumour growth	Vehicle, PD-1 ICB, [²²⁵Ac]Ac-FAPI-46 alone (different cycles)	PD-L1↑, CD8 + T cells↑	Well tolerated up to 3 × 60 kBq
2024	Poty et al.	[¹⁷⁷Lu]Lu-DOTA-4AH29 + pretargeting (4AH29-TCO + [¹⁷⁷Lu]Lu-DOTA-PEG7-tetrazine)	β-emitter, single-domain antibody, click chemistry	Pancreatic ductal adenocarcinoma (PDAC) PDX	88	Weekly for 3 weeks	Extended retention with pretargeting	Higher tumour absorbed dose 37.54 cGy/MBq, lower kidney absorbed dose 15.64 cGy/MBq	Prolonged survival, reduced kidney toxicity with pretargeting	[¹⁷⁷Lu]Lu-DOTA-4AH29, [^177^Lu]Lu-DOTA-PEG7-tetrazine	N/A	Reduced kidney toxicity, transient animal weight decrease
2024	Poplawski et al.	^177^Lu/^225^Ac/^161^Tb-PNT6555	α, β, auger electron-emitter, boronic acid derivative N-(pyridine-4-carbonyl)-D-Ala-boroPro	HEK-293-FAP xenografts	60 MBq for ^177^Lu/^161^Tb-PNT6555; 50 kBq for ^255^Ac-PNT6555	Single dose	Accumulation over 168 h	N/A	^225^Ac- and ^161^Tb- PNT6555 producing 80% and 100% survival	Vehicle, unlabeled precursors	N/A	No toxicity
2024	Mukkamala et al.	[¹⁷⁷Lu]Lu-FAP8-PEG3-IP-DOTA	β-emitter, albumin-binding	4T1 syngeneic tumour model, HT29, MDA-MB-231, KB xenografts	37	Single dose	3.4 ± 1 %ID/g at 168 h p.i.	1140 mGy/MBq (tumour), 220 mGy/MBq (kidney)	93% (MDA-MB-231), 65% (KB), 75% (HT29)	Vehicle	N/A	No significant toxicity but 5%-10% body weight loss during the first week
2024	Lindeman et al.	[¹⁷⁷Lu]Lu-FAP6-19	β-emitter, albumin-binding	4T1 syngeneic tumour model	N/A	Single dose	Improved tumour retention with optimized linker at 120 h p.i. (3%-4%ID/g)	569 mGy/MBq (tumour)	N/A	Vehicle	N/A	N/A
2024	Huang et al.	[¹⁷⁷Lu]Lu-FAPT	β-emitter, GlcP-PEG_2_ linker	A549-FAP lung cancer xenografts	37	Single dose	26.7 ± 6.8%ID/g at 4 h p.i.	N/A	Stronger tumour growth inhibition vs. [¹⁷⁷Lu]Lu-FAPI-04	Vehicle, 37 Mbq [^177^Lu]Lu-FAPI-04 treated group	N/A	No toxicity
2024	Hisada et al.	[²¹¹At]At-FAPI1, [²¹¹At]At-FAPI2	α-emitter, different length of PEG linker	BxPC3 xenografts	^211^At-FAPI1 (712.54 *±* 26.20 kBq); ^211^At-FAPI2 (731.80 *±* 33 kBq)	Single dose	2.39 ± 0.87 (FAPI1), 3.61 ± 0.63 (FAPI2) %ID/g (tumour) at 24 h p.i.	N/A	Both show tumour growth inhibition early, but Increase in tumour growth in all groups one month later	Vehicle	N/A	N/A
2024	Galbiati et al.	[^177^Lu]Lu-OncoFAP-23	β-emitter, trimerization of OncoFAP	SK-RC-52.hFAP xenografts or CT-26.hFAP syngeniec tumour model	30	Single dose	16%ID/g at 96 h p.i	N/A	Significant tumour inhibition for [^177^Lu]Lu-OncoFAP-23 treatment group; complete remission in all animals treated by combining with tumour-targeted interleukin 2	[^177^Lu]Lu-OncoFAP, [^177^Lu]Lu-BiOncoFAP, combine [^177^Lu]Lu-OncoFAP-23 and tumour-targeted interleukin 2	N/A	No toxicity
2024	Bendre et al.	[¹⁷⁷Lu]Lu-SB03178	β-emitter, benzo[h]quinoline-based	HEK293T: hFAP xenografts	27 MBq	Single dose	3.41 ± 0.29%I.A./g at 120 h p.i.)	9.22E02 mGy/MBq	N/A	N/A	N/A	No toxicity
2024	Abe et al.	[²¹¹At]At-FAPI1	α-emitter, PEG linker	MDA-MB-231 TNBC xenografts	1.04 ± 0.10	Single dose	2.70%ID/g (3 h)	N/A	Significant tumour inhibition	Vehicle	N/A	weight slightly decrease
2023	Zhong et al.	[¹⁷⁷Lu]Lu-(FAPI-04)₂	β-emitter, dimeric FAPI	SKOV3, A431, H1299 xenografts	7.4	2 injections	1.47 ± 0.15%ID/g at 24 h p.i.	N/A	Significant tumour inhibition	Vehicle	N/A	No toxicity
2023	Yang et al.	[¹⁷⁷Lu]Lu-LuFL ([¹⁷⁷Lu]21)	β-emitter, SiFA-based theranostic	HT-1080-FAP xenografts	24.05	Single dose	2.98 ± 0.98% ID/g at 24 h p.i.	N/A	Greater tumour inhibition than [¹⁷⁷Lu]Lu-FAPI-04	Vehicle, [ ^177^Lu]Lu-FAPI-04 treated group	N/A	No toxicity
2023	Xu et al.	[¹⁷⁷Lu]Lu-DOTA-NCS-PKU525	β-emitter, antibody-based	HT-1080-FAP xenografts	11.1	Single dose	19.02 ± 5.90%ID/g at 240 h p.i.	N/A	100% complete tumour suppression	Vehicle	N/A	No toxicity
2023	Song et al.	[²²⁵Ac]Ac-DOTA-PKU525	α-emitter, antibody-based	4T1-hFAP xenografts	0.0111	Single dose	12.44 ± 5.42%ID/g at 240 h p.i.	4.79E + 01 mGy/MBq (highest effective dose)	Significant tumour inhibition	Vehicle	N/A	No toxicity
2023	Pang et al.	[¹⁷⁷Lu]Lu-DOTA-4P(FAPI)_4_	β-emitter, tetrameric FAPI	HT-1080-FAP, U87MG xenografts	29.6	Single dose for HT-1080-FAP model, 3 doses for U87MG model	14.8 ± 0.9%ID/g at 96 h p.i.	N/A	Strong tumour inhibition vs. dimer/monomer	Vehicle, dimer, monomer	N/A	No toxicity
2023	Millul et al.	[¹⁷⁷Lu]Lu-FAPI-46-F1D, [¹⁷⁷Lu]Lu-FAPI-46-EB, [¹⁷⁷Lu]Lu-FAP-2286	β-emitter, dimer, albumin-binding, cyclic peptide	HT-1080.hFAP (low human FAP expression), HEK-293.hFAP (high human FAP expression)xenografts	0.8-1.0 MBq for biodistribution	Single dose	after 72 h, only the [ ^177^Lu]Lu-FAPI-46-EB retained highly in the tumour (83%)	6.76 and 11.3 mGy/MBq for [¹⁷⁷Lu]Lu-FAP-2286 and [¹⁷⁷Lu]Lu-FAPI-46-EB respectively in high FAP-expressing tumour	N/A	monomer FAPI-46 versus its dimer (FAPI-46-F1D), two albumin binders conjugates (FAPI-46-Ibu and FAPI-46-EB and cyclic peptide FAP-2286	N/A	No toxicity reported but Increased tumour-to-kidney ratio with FAP-2286
2023	Liu et al.	[²¹³Bi]Bi-FT-FAPI	α-emitter, linked with ammoniomethyl-BF3 (AMBF3)	HT-1080-FAP xenografts	3.7	6 injections	5.27 ± 1.58%ID/g at 8 h p.i.	N/A	Middle dose and high dose group showed stable tumour growth suppression, low dose group relapsed later	Vehicle	N/A	No toxicity
2023	Dekempeneer et al.	[²²⁵Ac]Ac-DOTA-4AH29, [¹³¹I]I-GMIB-4AH29	α and β-emitter, single-domain antibody	U87MG glioblastoma xenografts	38.60 ± 0.36 MBq of [^131^I]I-GMIB-4AH29, 20.43 ± 0.48 kBq of [^225^Ac]Ac-DOTA-4AH29	6 times over 3 weeks	5.93 ± 1.00%ID/g ([^131^I]I-GMIB-4AH29) and 3.68 ± 0.56%ID/g ([^225^Ac]Ac-DOTA-4AH29) at 72 p.i.	0.66 Gy/MBq ([^131^I]I-GMIB-4AH29); 7.84 Gy/0.1 MBq([^225^Ac]Ac-DOTA-4AH29)	Significant tumour suppression	Vehicle	N/A	Kidney toxicity with ([^225^Ac]Ac-DOTA-4AH29
2023	Aso et al.	[²¹¹At]At-FAPI1 to 5	α-emitter, comparison of linker PEG and PIP	PANC-1 xenografts	0.96 *±* 0.06 MBq (^211^At-FAPI5), 0.97 *±* 0.08 MBq (^211^At-FAPI1)	Single dose	Longer retention with PEG linker, with a tumour retention of 3.04 ± 0.69%ID at 3 h p.i.	N/A	No tumour regression but show clear growth inhibitory effect compared to the control group	Vehicle	N/A	No obvious toxicity
2022	Zhao et al.	[¹⁷⁷Lu]Lu-DOTA-2P(FAPI)_2_	β-emitter, dimeric FAPI	HT-1080-FAP CDXs, HCC-PDX	29.6	Single dose	Improved retention, with 2.85 ± 1.32%ID/g at 48 p.i.	N/A	Stronger tumour suppression vs. [¹⁷⁷Lu]Lu-FAPI-46	Vehicle, [¹⁷⁷Lu]Lu-FAPI-46	N/A	No toxicity
2022	Zhang et al.	[¹⁷⁷Lu]Lu-FAPI-C12, [¹⁷⁷Lu]Lu-FAPI-C16	β-emitter, albumin-binding	HT-1080-FAP xenografts	29.6	Single dose	6.50%ID/g ([¹⁷⁷Lu]Lu-FAPI-C16), 2.62 ± 0.65%ID/g [¹⁷⁷Lu]Lu-FAPI-C12 at 72 h p.i.	N/A	Significant tumour suppression	Vehicle, [¹⁷⁷Lu]Lu-FAPI-04	N/A	No toxicity
2022	Zboralski et al.	[¹⁷⁷Lu]FAP-2286	β-emitter, cyclic peptide	HEK293-FAP, sarcoma PDX	60	Single dose	Prolonged retention, with 16.4%ID/g at 72 h p.i.	2.8 Gy/MBq for [¹⁷⁷Lu]FAP-2286; 0.3 Gy/MBq for [^177^Lu]Lu-FAPI-46	113% tumour growth inhibition	Vehicle, nonradioac tive ^nat^Lu-FAP-2286, ^177^Lu-FAPI-46	N/A	No toxicity reported
2022	Xu et al.	[¹⁷⁷Lu]Lu-TEFAPI-06, [¹⁷⁷Lu]Lu-TEFAPI-07	β-emitter, albumin-binding	Pancreatic cancer PDX	3.7	3 injections	7.33 ± 2.28%ID/g and 7.57 ± 2.68%ID/g for ^177^Lu-TEFAPI-06 and ^177^Lu-TEFAPI-07 at 96 h p.i.	N/A	Significant tumour growth inhibition	Vehicle, [¹⁷⁷Lu]Lu-FAPI-04	N/A	No toxicity
2022	Wen et al.	[¹⁷⁷Lu]Lu-EB-FAPI-B1	β-emitter, Evans blue-modified	U87MG glioblastoma xenografts	30	Single dose	12.42 ± 1.54%ID/g at 96 h p.i.	N/A	Significant tumour suppression	Vehicle, [¹⁷⁷Lu]Lu-FAPI-02	N/A	N/A
2022	Meng et al.	[¹⁷⁷Lu]FSDD0I, FSDD1I, FSDD3I	β-emitter, albumin-binding	HCC-PDX xenografts	30	Single dose	~ 5% %ID/g at 96 h p.i.	N/A	N/A	Vehicle	N/A	N/A
2022	Liu et al.	[¹⁷⁷Lu]FAPI-46, [²²⁵Ac]FAPI-46	α and β-emitter, small molecule	PANC-1 xenografts	30 MBq (¹⁷⁷Lu), 30 kBq (²²⁵Ac)	Single dose	Rapid clearance, ~ 0.1%ID/g at 24 h p.i.	N/A	Significant tumour suppression	Vehicle	N/A	Body weight decrease
2021	Ma et al.	[¹³¹I]I-FAPI-02, [¹³¹I]I-FAPI-04	β-emitter, iodine-labeled	U87MG glioblastoma xenografts	5	Single dose	~ 30% ([¹³¹I]I-FAPI-04), ~ 10% ([¹³¹I]I-FAPI-02) %ID at 4 h p.i.	N/A	Strong tumour inhibition with intratumoural injection	Vehicle and Na^131^I group	N/A	No significant toxicity
2020	Watabe et al.	[²²⁵Ac]FAPI-04	α emitter, small molecule	PANC-1, MIA PaCa-2 xenografts	0.034	Single dose	0.097 ± 0.008%ID/g at 24 h p.i.	5.68 ± 0.77 Gy/MBq	Significant tumour suppression	Vehicle	N/A	No significant toxicity

*%ID* Percentage injected dose; *N/A* Not available; *p.i.* post injection

**Table 2:** Clinical studies of FAP targeted radionuclide therapy in last five years (2020–2025)

Year	Author	Clinical trial	Radiopharmaceuticals	Tumor type	Injected radioactivity	Dosimetry	Number of patients	Detailed response	ORR (CR + PR)	Survival outcome (months)	Adverse event
2025	Fu et al.	NCT05963386 (Phase II)	[¹⁷⁷Lu]Lu-LNC1004	Advanced metastatic cancers (11 types)	3.33 GBq/cycle (every six week)	4.69 ± 3.83 Gy/GBq (range: 1.18–25.03 Gy/GBq)	28	21.4% PR, 46.4% SD, 32.1% PD (best response in 23 evaluable patients)	21.4%	PFS: 4.0 (0.8–7.2); OS: 6.3 (0.8–11.7) Median (95% CI)	Grade 3/4 hematotoxicity in 21%; no grade 3/4 hepatotoxicity or nephrotoxicity
2024	Xie et al.	NR	[^177^Lu]Lu-FAP-2286	Advanced lung cancer	7.4 GBq/cycle (at least two cycles)	NR	9	44% PR, 33.3% SD, 22.2% PD	44%	PFS: 6.33 ± 2.45 (range 4–10); OS: 10.00 ± 3.12 (range 7–16)	No Grade 3/4 toxicity, fibrinogen reduction
2024	Helisch et al.	NR	[^213^Bi]Bi-FAPI-46	Metastatic solid tumors (3 colon cancer, 1 anal cancer, 1 breast cancer, and 1 prostate cancer)	1.6 GBq ( fractionated into 53 single applications (range, 5–12 RPT applications per patient; mean, 8.8 applications)	NR	6	16.7% PR, 16.7% SD, 66.7% PD	16.7%	NR	No major side effects
2024	Hamacher et al.	NR	[^90^Y]Y-FAPI-46	Sarcoma (Solitary Fibrous Tumor)	A total of 34 cycles (median, 3 cycles [IQR, 2 cycles])	2.9 Gy/GBq (IQR, 3.9 Gy/GBq; range, 0.5–24.7 Gy/GBq)	11	27% PR, 55% SD, 18% PD	27%	PFS: 7.5 (IQR, 7.3)	No severe toxicity
2024	Banihashemian et al.	NR	[^177^Lu]Lu-FAPI-2286	Metastatic mediastinal sarcoma	23 GBq in total (4 cycles)	NR	1	Partial response	NR	NR	Well tolerated
2024	Banihashemian et al.	NR	[^177^Lu]Lu-FAPI-2286	Advanced metastatic sarcoma	6.66–7.4 GBq (4 cycles, 6–8 weeks apart)	NR	8	50% PR, 37.5% SD, 12.5% PD	50%	OS: 7.8 ± 0.9	No Grade 3/4 toxicity, well tolerated, improved physical capacity and pain reduction
2023	Fu et al.	NCT05410821	[^177^Lu]Lu-EB-FAPI (^177^Lu-LNC1004)	Metastatic radioiodine-refractory thyroid cancer (mRAIR-TC)	2.22/3.33/4.99 GBq	8.50 ± 12.36 Gy/GBq	12	25% PR, 58% SD, 17% PD	25%	NR	Some Grade 3/4 hematotoxicity, well tolerated
2022	Kaghazchi et al.	NR	[^177^Lu]Lu-FAPI-46	End-stage metastatic pancreatic adenocarcinoma (PADC)	1.85 GBq	NR	1	Transient pain relief, but disease progression	NR	Patient deceased after 45 days	No significant toxicity reported
2022	Fu et al.	NCT04849247	[^177^Lu]Lu-FAPI-46	Metastatic nasopharyngeal carcinoma	3.7 GBq	0.026 mGy/MBq (whole body), 0.886 mGy/MBq (kidney), 0.136 mGy/MBq (liver)	1	Mixed response: tumor regression in ribs and thoracic vertebrae, but progression in abdominal lymph nodes, liver, skull, and pelvis	NR	NR	NR
2022	Fu et al.	NCT04849247	[^177^Lu]Lu-FAPI-46	Radioiodine-refractory differentiated thyroid cancer (RAIR-DTC)	5.55 GBq (4 cycles, 8 weeks apart), (cumulative dose: 16.7 GBq)	NR	1	Stable disease after 4 cycles, no significant tumor shrinkage, pain relief, ECOG improved (1 → 0)	NR	NR	Well tolerated
2022	Ferdinandus et al.	NR	[^90^Y]Y-FAPI-46	Advanced-stage solid tumors (sarcoma, pancreatic cancer)	3.8 GBq (IQR, 3.25–5.40 GBq) for the first cycle and 7.4 GBq (IQR, 7.3–7.5 GBq) for any subsequent cycle	Tumor: median 1.28 (0.83–1.71); Gy/GBq Kidney: 0.52 Gy/GBq; Bone marrow: 0.04 Gy/GBq	9	50% SD, 50% PD	0%	PFS: 18.5 d (IQR, 14.8–38.5 d)	grade 3 thrombocytopenia (*n* = 4), grade 3 anemia (*n* = 1), ≥grade 3 new increases of hepatic or pancreatobiliary serum markers
2022	Fendler et al.	NR	[^90^Y]Y-FAPI-46	Advanced sarcoma, pancreatic cancer, prostate, gastric cancer	3.7–7.4 GBq per cycle, up to 4 cycles	Tumor: 2.81 Gy/GBq; Kidney: 0.53 Gy/GBq, Bone marrow: 0.04 Gy/GBq	21	1 PR (5%), 7 SD (33%), Disease control rate: 38%	5%	PFS: 3.4 (1.1–5.7) [median (95% CI)]; OS: 10.0 (4.4–15.5)	Grade 3 or 4 anemia (6/21, 29%) and thrombocytopenia (6/21, 29%)
2022	Baum et al.	NR	[^177^Lu]Lu-FAP-2286	Advanced adenocarcinomas of the pancreas, breast, rectum, or ovary	(5.8 ± 2.0 GBq; range, 2.4–9.9 GBq)	Tumor: 3.0 ± 2.7 Gy/ GBq; Kidneys: 1.0 ± 0.6 Gy/GBq; red marrow: 0.05 ± 0.02 Gy/GBq	11	18% SD, 82% PD	0%	NR	Grade III hematotoxicity (*n* = 3)
2022	Barashki et al.	NR	[^177^Lu]Lu-FAPI-46	Multiple Endocrine Neoplasia Type 2 A (MEN2A) with metastatic paraganglioma, medullary thyroid carcinoma, pheochromocytoma	7.4 GBq	NR	1	Abdominal pain relief	NR	NR	No significant toxicity reported
2022	Ballal et al.	NR	[^177^Lu]Lu-DOTAGA.(SA.FAPi)2	Radioiodine-refractory differentiated thyroid cancer (RR-DTC)	8.2 ± 2.7 GBq (range 5.5–14 GBq)	1.08E + 01 (IQR: 4.16E + 00 to 8.97E + 01) mSv/MBq per cycle	15	26.7% PR, 20% SD Tg levels had decreased in all patients	26.7%	NR	No Grade 3/4 hematological, renal, or hepatotoxicity
2021	Rathke et al.	NR	[^90^Y]Y-FAPI-46	Metastatic breast and colorectal cancer	7.4 GBq (initial), cumulative 28.1 GBq over 4 cycles	NR	1	Stable disease in breast cancer metastases, remission of colorectal cancer metastases, progression after 7 months	NR	NR	No severe toxicity, patient died 4 months post-treatment due to BC progression
2021	Kuyumcu et al.	NR	[^177^Lu]Lu-FAPI-04	Advanced metastatic cancer (breast cancer, thymic carcinoma, papillary thyroid can cer, metastatic ovarian carcinosarcoma)	Low-dose (267.5 ± 8.6 MBq (range, 259–278 MBq)	0.62 (bone metastases), 0.38 (metastatic lymph nodes, 0.33 (liver metastases), 0.37 (metastatic soft tissue), 0.25 (kidney), 0.11 (liver), 0.04 (bone marrow) mGy/MBq	4	NR	NR	NR	No significant toxicity, allows high dose administration
2021	Kratochwil et al.	NR	[^153^Sm]Sm-FAPI-46, [^90^Y]Y-FAPI-46	Lung metastases of soft tissue sarcoma	Cumulative 20 GBq (^153^Sm) + 8 GBq (^90^Y) over 3 cycles	NR	1	Stable disease for 8 months	NR	NR	Well tolerated, no severe toxicity reported
2021	Ballal et al.	NR	[^177^Lu]Lu-DOTA.SA.FAPi, [^177^Lu]Lu-DOTAGA.(SA.FAPi)_2_	Various advanced cancers	^177^Lu]Lu-DOTA.SA.FAPi: 2.96 GBq (single cycle); [^177^Lu]Lu-DOTAGA.(SA.FAPi)_2_: 1.48 GBq (3 cycles)	[^177^Lu]Lu-DOTA.SA.FAPi: 0.603 (0.230–1.810) Gy/GBq, [^177^Lu]Lu-DOTAGA.(SA.FAPi)_2_: 6.70 (3.40–49) Gy/GBq	10	[^177^Lu]Lu-DOTA.SA.FAPi: 3 patients demonstrated replapse in the clinical symptons; [^177^Lu]Lu-DOTAGA.(SA.FAPi)_2_: 7 patients demonstrated a clinical response	NR	NR	Well tolerated, 1 patient had Grade III anemia, no other severe toxicity
2021	Ballal et al.	NR	[^177^Lu]Lu-DOTA.SA.FAPi	End-stage breast cancer	3.2 GBq (single cycle)	Tumor: 1.48 mGy/MBq, Brain metastasis: 3.46 mGy/MBq	1	Reduction in headache intensity	NR	NR	No treatment-related adverse events
2021	Assadi et al.	NR	[^177^Lu]Lu-FAPI-46	Various advanced cancers	1.85–4.44 GBq per cycle, total injected activity from 1.85 to 13.7 GBq	0.026 (whole body), 0.136 (liver), 0.886 (kidney), 0.02 (spleen) mGy/MBq	21	14.3% PR, 57.1% SD, 28.6% PD	14.3%	PFS: 9.0 (range, 5.0–30.0) OS: 25 (range, 6.0-110.0)	Mostly well tolerated, 1 case of Grade 3 anemia, grade 1 thrombocytopenia, and grade 1 leukopenia

*NR* No report; *PR* Partial response; *SD* Stable disease; *PD* Progressive disease; *ORR* Objective response rate (sum of PR + CR, expressed as a percentage of total evaluable patients)

### Development and evaluation of small molecule FAP inhibitors

Over the past five years, significant advances have been made in the field of FAP-TRT, with a strong focus on modifying the targeting moiety or the radionuclide and evaluating their anticancer efficacy in tumour animal models. Among small molecule derivatives, FAP inhibitors (FAPIs) such as FAPI-02, FAPI-04, and FAPI-46 have demonstrated promising tumour retention and have been radiolabelled with β-emitters, including lutetium-177 and iodine-131, as well as α-emitters, such as actinium-225 and bismuth-213, for evaluation across various tumour models. Studies in pancreatic and glioblastoma xenografts consistently showed that radiolabelled FAPI-46 exhibited prolonged tumour retention (6–24 h post-injection, p.i.) and marked tumour suppression as compared to FAPI-02 and FAPI-04 [39–41]. Additionally, Liu et al. reported a transient weight loss following both [¹⁷⁷Lu]Lu-FAPI-46 and [²²⁵Ac]Ac-FAPI-46. In mice treated with [¹⁷⁷Lu]Lu-FAPI-46, weight loss was observed in the 10 MBq and 30 MBq groups, whereas in the [²²⁵Ac]Ac-FAPI-46–treated cohorts (3 kBq, 10 kBq, and 30 kBq), all groups showed a reduction in body weight, with recovery occurring after day 7 in the 3 kBq and 10 kBq groups only [40]. More recently, [¹⁷⁷Lu]Lu-SB03178, a benzo[h]quinoline-based ligand, demonstrated improved tumour retention time of up to 120 h p.i. — approximately five times longer than that of [^177^Lu]Lu-FAPI-46 — as well as dose delivery compared to earlier derivatives, supporting its clinical potential [41].

### Optimising tumour retention through polyethylene glycol (PEG) linkers, organotrifluoroborate linkers, SiFA-based linkers, and albumin-binding strategies

Since the rapid washout of the first generation of FAPi-based radiopharmaceuticals from tumours limited their anti-cancer efficacy, several studies have explored conjugating the compounds with albumin-binding moieties to prolong their blood circulation, such as Evans blue, 4-(p-iodophenyl) butyric acid, and fatty acids, to extend their circulation time in the bloodstream [42–48], though at the cost of increased off-target uptake in liver, kidneys, and lungs [45, 47]. PEG linkers have also been incorporated to extend tumour residence time. For instance, [^211^At]At-FAPI derivatives with PEG linkers exhibited improved tumour uptake and tumour growth suppression in xenograft models, although effects on tumour regression remained limited [49–51]. Huang et al. developed a GlcP-PEG2 modified [^177^Lu]Lu-DOTA-FAPT that outperformed [^177^Lu]Lu-FAPI-04 in uptake and survival benefit in FAP + tumour models [50]. Other linker innovations include the AMBF3 organotrifluoroborate linker for [^213^Bi] labelling, which showed potent antitumour efficacy [52], and the SiFA/DOTAGA dual-labelling platform, where [^177^Lu]LuFL achieved strong tumour inhibition and prolonged retention [53].

### Multimerization, heterodimers approaches, cyclic peptides, boronic acid-based and covalent sulfur(VI) fluoride exchange (SuFEx)-engineered ligand for improved tumour uptake

To increase tumour retention and uptake, various ligand engineering strategies have been developed. Among them, a multimerisation method was developed to modify the ligand to prolong the tumour retention time. Dimeric and tetrameric [^177^Lu]Lu-FAPI ligands demonstrated improved retention and efficacy in high FAP-expressing tumours, though they also increased uptake in kidneys and liver [54, 55]. Alternative ligand designs such as the cyclic peptide FAP-2286 and boronic acid-based PNT6555 showed excellent tumour-to-background contrast and potent therapeutic effects, especially when labelled with α- or β-emitters [56, 57].

Beyond single-target optimisation, heterodimeric tracers that combine FAP-targeting moieties with ligands for other tumour-associated targets—such as PSMA or integrin αvβ3—have been developed to broaden tumour coverage and enhance uptake [58, 59]. For example, [^177^Lu]Lu-FAP-RGD, designed by linking FAP-2286 with a cyclic arginine–glycine–aspartic acid (RGD) peptide to simultaneously target FAP and αvβ3 integrin, achieved significant antitumour effects in preclinical glioblastoma models [59]. However, these approaches often still rely on reversible binding mechanisms, which can lead to limited tumour retention.

To address this limitation, a novel strategy based on covalent engineering using SuFEx chemistry has recently emerged [60]. By installing SuFEx warheads on FAPI molecules, covalent targeted radioligands (CTR-FAPIs) can irreversibly bind to FAP in tumours. Among them, FAPI-mFS, using a meta-fluorosulfate warhead, achieved a 257% increase in tumour uptake and 13-fold higher tumour retention in mice compared to conventional FAPI-04. Importantly, this SuFEx-engineered FAP, radiolabelled with β emitter, achieved complete tumour regression in mice.

However, the use of xenograft models made of artificially overexpressed FAP tumour cells raises concerns about translatability due to the absence of CAF-tumour interactions and the artificially elevated expression levels compared to human tumours, which will be discussed in more detail in a later section.

### Radiolabelled antibody-based approaches for FAP targeting

Biologics such as antibodies, engineered fragments or other immune-constructs represent another key class of FAP-targeted therapeutics. Particularly, radiolabelled FAP-targeting antibodies have gained increasing interest for their excellent affinity and retention properties in TRT, offering enhanced tumour selectivity. Early examples such as [^131^I]I-sibrotuzumab and [^177^Lu]Lu-ESC11 demonstrated high tumour uptake and therapeutic potential [61–63], though their long circulation time may result in higher absorbed doses in non-target organs such as spleen and liver. Recent advances in antibody-based FAP-TRT have led to the development of both full-length antibodies, such as PKU525 [64, 65], and single-domain antibodies, such as 4AH29 [66]. When labelled with [²²⁵Ac] or [¹⁷⁷Lu], both constructs showed potent antitumour efficacy. Notably, Song et al. reported that treatment with [²²⁵Ac]Ac-DOTA-PKU525 achieved significant tumour inhibition without observable toxicity [65], whereas Dekempeneer et al. observed renal toxicity following therapy with [²²⁵Ac]Ac-DOTA-4AH29 [66]. These findings highlight the impact of antibody format on biodistribution and safety profiles in FAP-TRT. To further address the nephrotoxicity associated with small antibody fragments, pre-targeting strategies using a FAP-targeting single-domain antibody (4AH29) functionalized with trans-cyclooctene and subsequent administration of [^177^Lu]Lu-DOTA-PEG7-tetrazine via click chemistry have further improved tumour selectivity while mitigating nephrotoxicity [67].

### Enhancing FAP-TRT with ICIs

Beyond focusing on ligand modification to prolong the tumour retention time, several studies have also investigated combination strategies, such as integrating FAP-TRT with ICIs (e.g., PD-L1/PD-1 blockade), which resulted in improved tumour control and immune modulation. Studies in immunocompetent models demonstrated that [^225^Ac]Ac-FAPI-46 [68], [^225^Ac]Ac-DOTA-4AH29 [15], [^177^Lu]Lu-DOTA-2P(FAPI)_2_ [69] and [^177^Lu]Lu -LNC1004 [70] sensitised tumour to ICI, thereby improving responsiveness, reversing resistance, and inducing durable responses, thereby providing a strong biological rationale for synergistic combination strategies.

More specifically, Taddio et al. [68] investigated [²²⁵Ac]Ac-FAPI-46, administered either as monotherapy or in combination with PD-1 blockade, in fibrosarcoma models (FSA) bearing either endogenous FAP-expressing cells or FAP-overexpressing (FSA-F) cells, enabling direct comparison between low- and high-FAP tumours. In endogenous FAP-expressing tumours, [²²⁵Ac]Ac-FAPI-46 monotherapy showed limited efficacy, whereas combination therapy achieved tumour growth delay in 55% (6/11) and partial regression in 18% (2/11) of mice. In FSA-F tumours with enforced FAP overexpression, both [²²⁵Ac]Ac-FAPI-46 and PD-1 blockade individually exerted tumour-inhibitory effects, and their combination did not provide further benefit, likely reflecting the already high sensitivity of this model to PD-1 inhibition. In contrast, in immunologically cold, ICI-resistant FSA-F tumours, the combination of [²²²⁵Ac]Ac-FAPI-46 and PD-1 blockade restored responsiveness to ICI, resulting in complete tumour regression and durable tumour-free survival for up to 52 days in 56% (5/9) of treated mice. These findings suggest that synergy between FAP-TRT and ICIs may be particularly relevant in tumours with lower or heterogeneous FAP expression, where radioligand-induced immune remodelling is required to enable effective ICI responses.

Similarly, Ceuppens et al. [15] demonstrated that [²²⁵Ac]Ac-DOTA-4AH29 not only prolonged survival but also increased PD-L1 expression in tumour cells and PD-1 and LAG3 expression on CD8⁺ T cells in an immunocompetent lung cancer model. Subsequent PD-L1 blockade further enhanced tumour regression, although increased weight loss was observed, indicating that potential toxicity requires careful optimisation.

In colorectal cancer models, Chen et al. [69] showed that [¹⁷⁷Lu]Lu-DOTA-2P(FAPI)₂ induced DNA double-strand breaks and upregulated PD-L1, promoting antitumour immune activation. While monotherapy achieved substantial tumour suppression, its combination with PD-L1 blockade led to complete tumour eradication in 100% of treated mice, accompanied by long-lasting immunological memory upon tumour rechallenge. Likewise, Zhao et al. [70] reported that [¹⁷⁷Lu]Lu-LNC1004 increased PD-L1 expression, CD8⁺ T-cell infiltration, M1-type macrophages, and antigen presentation pathways in CAF-containing tumour co-culture models. Combination therapy induced complete responses in all MC38/NIH3T3-FAP-bearing mice, though only 37.5% (3/8) complete remission was achieved in CT26/NIH3T3-FAP tumours, indicating that tumour immune phenotype influences treatment responsiveness. Multi-omics profiling (including scRNA-seq, TCR sequencing, and immune phenotyping) suggests that [^177^Lu]Lu-LNC1004 treatment is associated with increased antigen presentation, activating APC–T cell co-stimulatory networks (CD137, CXCL16–CXCR6, CCL5–CCR5), expanding CD8⁺ T cells, increasing TCR diversity, reprogramming myeloid cells, and promoting IRF1⁺ neutrophil infiltration, ultimately enabling complete tumour regression and durable immune memory. These immune remodelling effects may underlie the observed improvement in responsiveness to PD-L1 blockade; however, further mechanistic validation is required to determine causality.

In summary, these preclinical investigations have driven remarkable advances in FAP-TRT, with most efforts focused on chemical modifications to enhance tumour retention. However, this emphasis on ligand engineering often overshadows other critical determinants for clinical translation—notably the choice of biologically relevant models, the role of the immune system in shaping treatment outcomes, and the integration of radiobiological principles beyond dosimetry. These overlooked aspects become even more apparent when contrasting preclinical findings with emerging clinical evidence, a gap that will be addressed in the following section.

## Clinical outcomes and ongoing clinical trials

Over the past five years, early-phase clinical experience on FAP-TRT have demonstrated the feasibility and safety of FAP-TRT across multiple tumour types. However, most studies were conducted in small, heterogeneous patient cohorts and often under compassionate-use conditions, resulting in only moderate and variable therapeutic efficacy across advanced solid tumours, as summarised in Table 2. Agents such as [¹⁷⁷Lu]Lu-FAPI-46, [⁹⁰Y]Y-FAPI-46, [¹⁷⁷Lu]Lu-FAP-2286, and [²¹³Bi]Bi-FAPI-46 have been evaluated in diverse tumour types, including sarcomas, pancreatic cancer, thyroid cancer, lung cancer, and other metastatic malignancies. The median ORR across all published studies is only 21.4%, with most below 30% and some showing not even partial responses [16–36]. A few small-scale studies (e.g., [^177^Lu]Lu-FAPI-2286 in sarcoma, [^90^Y]Y-FAPI-46 in lung cancer) reported ORRs > 40% [17, 21], but these studies only involved < 10 patients and require cautious interpretation. Most other studies show partial response (PR) or stable disease (SD) as the main outcomes, with limited durability, underscoring the need for better patient selection and optimised treatment strategies. Besides, several case studies also reported mixed responses or transient improvements, often without complete tumour regression [20, 23–25, 29, 31, 35] and even became disease progression in a later stage [23, 31].

For reference, established TRT modalities such as somatostatin receptor subtype 2 (SSTR2)-targeted therapy (e.g., [^177^Lu]Lu-DOTATATE) [37] and PSMA-TRT (e.g., [^177^Lu]Lu-PSMA) [38] typically achieve higher response rates (ORRs) (often reaching 40–70% in selected populations). However, such a comparison should be interpreted with caution. Unlike SSTR2- or PSMA-targeted therapy, which requires well-defined molecular expression in patients with specific tumour types such as neuroendocrine tumours (NETs) and prostate cancer for treatment eligibility, FAP-TRT was initially developed and applied as a pan-tumour stromal targeting approach due to the broad activation of CAFs across diverse solid tumours. As a result, early clinical studies naturally involved highly heterogeneous tumour cohorts, which fundamentally limits comparability in response rates across TRT classes. Moreover, FAP-TRT primarily targets cancer-associated fibroblasts within the TME rather than tumour cells directly, and the choice of radionuclide (α- vs. β-emitter) may further influence therapeutic dynamics. Thus, both differences in biological mechanisms and differences in patient selection criteria reduce the relevance of direct numerical comparisons of response rates.

Most studies on FAP-TRT reported favourable safety profiles, with minimal grade 3/4 haematological, hepatic, or renal toxicities. Dosimetry data showed generally acceptable radiation absorbed doses to organs at risk such as kidneys and bone marrow, which are considered dose-limiting, partly due to non-negligible uptake in these normal tissues. For instance, the mean kidney dose in several studies ranged from 0.25 to 1.0 Gy/GBq [28, 32], while the bone marrow dose remained around or below 0.05 Gy/GBq [28].

Currently, at least 10 Phase I or I/II clinical trials are actively recruiting globally to further evaluate FAP-TRT, as listed in Table 3. These trials explore novel agents like [¹⁷⁷Lu]Lu-DOTA-EB-FAPI, [¹⁷⁷Lu]Lu-OncoFAP-23, [¹⁷⁷Lu]Lu-PNT6555, and [¹⁷⁷Lu]Lu-FAP-75 in various advanced FAP-positive malignancies. Notably, the majority of ongoing clinical trials are still in Phase I, primarily focusing on safety, dosimetry, and feasibility, rather than therapeutic efficacy, underscoring that FAP-TRT remains in an early clinical development stage. Once safety and dosing parameters are better defined, therapeutic optimisation will likely be addressed in subsequent phases. Importantly, preclinical studies have consistently shown that FAP-TRT can remodel the tumour stroma and enhance antitumour immune activation, thereby providing a strong biological rationale for integrating FAP-TRT into combination regimens, particularly with immune checkpoint blockade, in future therapeutic strategies.

**Table 3:** Ongoing clinical trials of FAP-Targeted radiopharmaceuticals (as of February 2025)

NCT number	Investigational drug / Intervention	Disease	Phase	Planned enrollment	Affiliation	Period	Status
NCT05410821	[^¹⁷⁷^Lu]Lu-DOTA-EB-FAPI	Metastatic radioactive Iodine Refractory Thyroid Cancer	Phase I	20	The First Affiliated Hospital of Xiamen University, China	15/06/2022-15/06/2025	Recruiting
NCT06081322	[^¹⁷⁷^Lu]Lu-EB-FAPI	Advanced pancreatic cancer and cholangiocarcinoma	Phase I	29	Zhejiang University, China	01/09/2023-30/06/2025	Recruiting
NCT06640413	[^¹⁷⁷^Lu]Lu-OncoFAP-23	Advanced/metastatic Fibroblast Activation Protein (FAP)-positive solid tumors	Phase I	56	Philogen S.p.A.	31/05/2025-31/12/2028	Not yet recruiting
NCT04939610	[^¹⁷⁷^Lu]Lu-FAP-2286	Pancreatic ductal adenocarcinoma, non-small cell lung cancer, and breast cancer	Phase I and II	222	Novartis Pharmaceuticals	30/07/2021-30/06/2028	Recruiting
NCT05723640	[^¹⁷⁷^Lu]Lu-LNC1004	Recurrent or metastatic, fibroblast activation protein-positive solid tumors	Phase I	24	Yantai LNC Biotechnology Singapore PTE. LTD.	03/10/2023-30/03/2025	Recruiting
NCT05432193	[^¹⁷⁷^Lu]Lu-PNT6555	Solid tumors that have FAP over-expression	Phase I	24	POINT Biopharma, a wholly owned subsidiary of Eli Lilly and Company	13/07/2022-12/2026	Recruiting
NCT06636617	[^¹⁷⁷^Lu]Lu-JH04	Patients with FAP-Positive Tumors	Phase I	9	First Affiliated Hospital of Fujian Medical University, China	21/08/2024-31/08/2025	Recruiting
NCT06197139	[^¹⁷⁷^Lu]Lu-XT117	Patients with FAP-positive advanced solid tumors	Phase I	20	The First Affiliated Hospital of Guangzhou Medical University, China	01/2024-12/2026	Recruiting
NCT06211647	[^¹⁷⁷^Lu]Lu-XT117	Patients with FAP-positive advanced solid tumors	Phase I	20	Chinese PLA General Hospital	01/2024-12/2026	Recruiting
NCT06553846	[^¹⁷⁷^Lu]Lu-FAP-75	Patients with advanced solid tumors	Phase I	50	Fudan University, China	19/08/2024-18/08/2025	Recruiting

All trial information is based on ClinicalTrials.gov records as of February 2025

## Challenges in the clinical applications of FAP-TRT

Given the limited efficacy data from early-phase clinical experience, further research is essential to address several key challenges in the clinical application of FAP-TRT. Multiple biological and technical factors may contribute to its suboptimal therapeutic outcomes:

1. Short tumour retention is a major limitation. Rapid clearance of radiopharmaceuticals from tumour tissue, as observed in studies such as those by Kaghazchi et al. [23] and Balla et al. [34], significantly reduces radiation dose delivery and hence therapeutic effiacy.
2. Interpatient variability in TME—particularly in FAP expression, perfusion, and stromal composition—can lead to heterogeneous radiopharmaceuticals uptake and inconsistent treatment responses. This heterogeneity complicates both patient selection and outcome prediction. Although most preclinical efforts are currently directed toward improving tumour retention time through tracer modifications and radionuclide optimisation, this alone may not be sufficient to overcome the therapeutic limitations of FAP-TRT. Therefore, complementary efforts should also aim to uncover the biological basis of treatment response, including FAP heterogeneity, stromal dynamics, and tumour immune interactions, which all play a fundamental role in determining clinical efficacy.
3. The lack of standardised dosimetry protocols may limit efforts toward dose optimisation, as it complicates the comparison of absorbed doses across studies and prevents the establishment of clear therapeutic dose thresholds. Studies have reported highly variable tumour absorbed doses (e.g., Fu et al. [16]: 4.7 ± 3.8 Gy/GBq), highlighting the need for harmonized and individualized dosimetry approaches.
4. While FAP-TRT is generally well tolerated, dose-limiting toxicities remain a consideration in higher-dose regime. Although kidneys are involved in clearance and receive notable radiation exposure, no grade 3/4 nephrotoxicity has been reported in clinical studies to date. In contrast, hematologic toxicity—particularly grade 3/4 anaemia and thrombocytopenia—has been observed in several studies and appears to be the main dose-limiting factor.

Additionally, biomarkers for patient selection and pharmacodynamic monitoring are still under development. Imaging parameters including SUV_max_, SUV_peak_ and tumour-to-background ratio (TBR) [21], have shown utility in response assessment. Reductions in serum tumour markers (e.g., thyroglobulin) [30] and clinical improvements (e.g., pain relief, ECOG performance) [25] have also been reported. However, there is currently no validated predictive biomarker to guide therapy or anticipate resistance, underlining the urgent need for translational research in this area.

To better realise the potential of FAP-TRT, future research could benefit from well-designed prospective trials that standardise treatment protocols, refine patient selection strategies, and assess long-term outcomes, such as overall survival and quality of life. While large-scale, randomised trials with extended follow-up periods remain the gold standard, they are often constrained by logistical, financial, and ethical challenges. Therefore, alternative strategies—such as multi-centre collaboration, adaptive trial design or real-world data collection—may offer more practical approaches to generate robust clinical evidence and guide the integration of FAP-TRT into routine practice. To avoid unnecessary resource expenditure and improve translational value, strategies such as patient-derived organoids, organoid-on-chip systems, and ex vivo tumour slices can be incorporated into early-stage research and clinical trial design. These models offer an opportunity to assess the practical applicability and therapeutic relevance of FAP-TRT in a more physiologically relevant context, helping to identify promising approaches and eliminate ineffective ones before large-scale investment, thereby improving the efficiency of clinical translation.

## Challenges and considerations in FAP-TRT translation

### Discrepancies in FAP expression levels, spatial distribution of FAP-expressing cells and complexity of TME between preclinical models and human tumours

One of the primary challenges in the development of FAP-TRT lies in the selection of appropriate tumour models, as current preclinical models often do not accurately reflect the FAP expression levels observed in human tumours.

Most preclinical studies regarding FAP-TRT rely on cell line-derived xenograft (CDX) tumour models, where FAP expression is artificially induced through transfection [14, 15, 40, 41, 43, 45, 46, 49–55, 57, 63–66, 71–77]. Among these, many employ sarcoma CDX models using the HT1080-FAP in which the tumour cells were transfected with human FAP [46, 52, 53, 55, 64], whereas sarcoma cells in patients were identified naturally expressing FAP [78]. This results in uniformly high FAP levels, which do not accurately reflect the heterogeneous FAP expression found in patient tumours. While patient-derived xenografts (PDX) have been employed in some studies [42, 44, 54, 56, 67], CAFs tend to diminish during serial passaging, leading to altered FAP expression and an incomplete TME [79, 80].

Already in 2003, Tahtis et al. [62] explored radiopharmaceuticals targeting FAP in the human skin/mouse chimeric model, which can retain human-derived CAFs, providing a more representative stromal environment. However, the conventional PDX models are typically established in immunocompromised mice, resulting in the absence of a functional immune system and the progressive loss or replacement of human CAFs during serial passaging. As a result, these models fail to capture the dynamic interaction between CAFs and immune cells, as well as the immune modulation induced by TRT that contributed to therapeutic efficacy. To better recapitulate the immune–tumour interactions and dynamic condition of TME, some studies have attempted to use syngeneic models [47, 48, 68, 69]. Meanwhile, co-culture systems incorporating tumour cells and CAFs have also been explored [70], but these often rely on genetically engineered CAFs overexpressing FAP, which fails to fully mimic the natural complexity of the TME.

Besides absolute FAP expression levels, the spatial distribution of FAP-positive cells within the TME also critically influences the therapeutic efficacy of FAP-targeted radiotherapy [81]. This CAF-cancer cell adjacency is critical for determining whether short-range α-emitters or longer-range β-emitters are more appropriate. In settings where CAFs and tumour cells are intermixed at close proximity, α-emitters may efficiently deliver high-LET damage. In contrast, when CAFs form larger stromal compartments or are spatially separated from tumour cells that do not themselves express FAP, β-emitters are more likely to benefit from the broader cross-fire irradiation range. However, the distribution of FAP-expressing cells in preclinical tumour models often differs significantly from that in human tumours. A study further demonstrated that FAP expression in CDX models using HT1080-huFAP cells was approximately two-fold higher than in U87 MG-derived xenografts, which recruit murine fibroblasts [82], indicating artificially elevated FAP expression in the genetically modified model. Moreover, for most epithelial-derived human tumours, FAP is mainly expressed in CAFs rather than tumour cells [8], meaning that these models in which tumour cells are engineered to express FAP do not recapitulate the native spatial organisation of FAP-positive stromal cells within the TME. Additionally, xenograft models derived from CDX lack endogenous and spontaneous CAFs [70] and tumour vasculature, limiting their ability to mimic real tumour conditions. As noted above, CAFs in PDX models are progressively replaced or depleted during serial passaging, which further limits their ability to recapitulate the native tumour-stroma architecture. This highlights the importance of utilising orthotopic models and syngeneic systems to better capture the complexities of tumour-immune interactions in FAP-TRT.

To our knowledge, nearly all preclinical studies investigating FAP-TRT have used subcutaneous tumour models rather than (spontaneous) orthotopic tumour models. This significantly limits translational relevance, as subcutaneous models lack the in situ TME, native vascular architecture, and metastatic potential seen in human cancers. The absence of a physiologically relevant TME may lead to an overestimation of FAP-TRT efficacy, failing to account for tumour heterogeneity, invasion dynamics, and metastatic progression. Given that patients receiving FAP-TRT often present with widespread metastases and advanced disease, models more reflective of the clinical situation, such as spontaneous orthotopic tumour models, are urgently needed. These models can provide a more physiologically relevant platform to: (a) Examine the role of heterogeneous CAF populations in tumour progression; (b) Investigate the combinatorial effects of ICIs and FAP-TRT in a more representative TME, for which humanised PDX models could serve as a promising alternative; (c) Assess tumour invasion and metastatic dissemination following FAP-TRT, rather than focusing solely on primary tumour regression. By integrating clinically relevant tumour models, future studies can better predict therapeutic responses and optimise FAP-TRT strategies for patients with aggressive and metastatic cancers.

Notably, the choice of preclinical model should be aligned with the specific research objective, while also considering the practical complexity of model establishment and the need to avoid unnecessary consumption of time, resources, and patient-derived materials. In early-phase studies, CDX and PDX models remain valuable for confirming molecular targeting, tracer biodistribution, and preliminary therapeutic effects. When addressing questions involving tumour–TME interactions, CAF-subtypes and FAP expression heterogeneity, dosing optimisation, or assessing synergistic combinations such as FAP-TRT with immunotherapy, models with intact immune systems, including syngeneic or humanised PDX platforms, should be prioritised. Finally, orthotopic tumour models play a critical role in the later preclinical phase before clinical translation, where they are useful to refine dose scheduling, evaluate treatment durability, and better predict clinical response and safety. Such a stepwise modelling strategy balances translational relevance with feasibility, allowing FAP-TRT research to progress efficiently from molecular validation to clinically meaningful applications.

### Heterogeneity of cafs: a double-edged sword

**Fig. 2:**
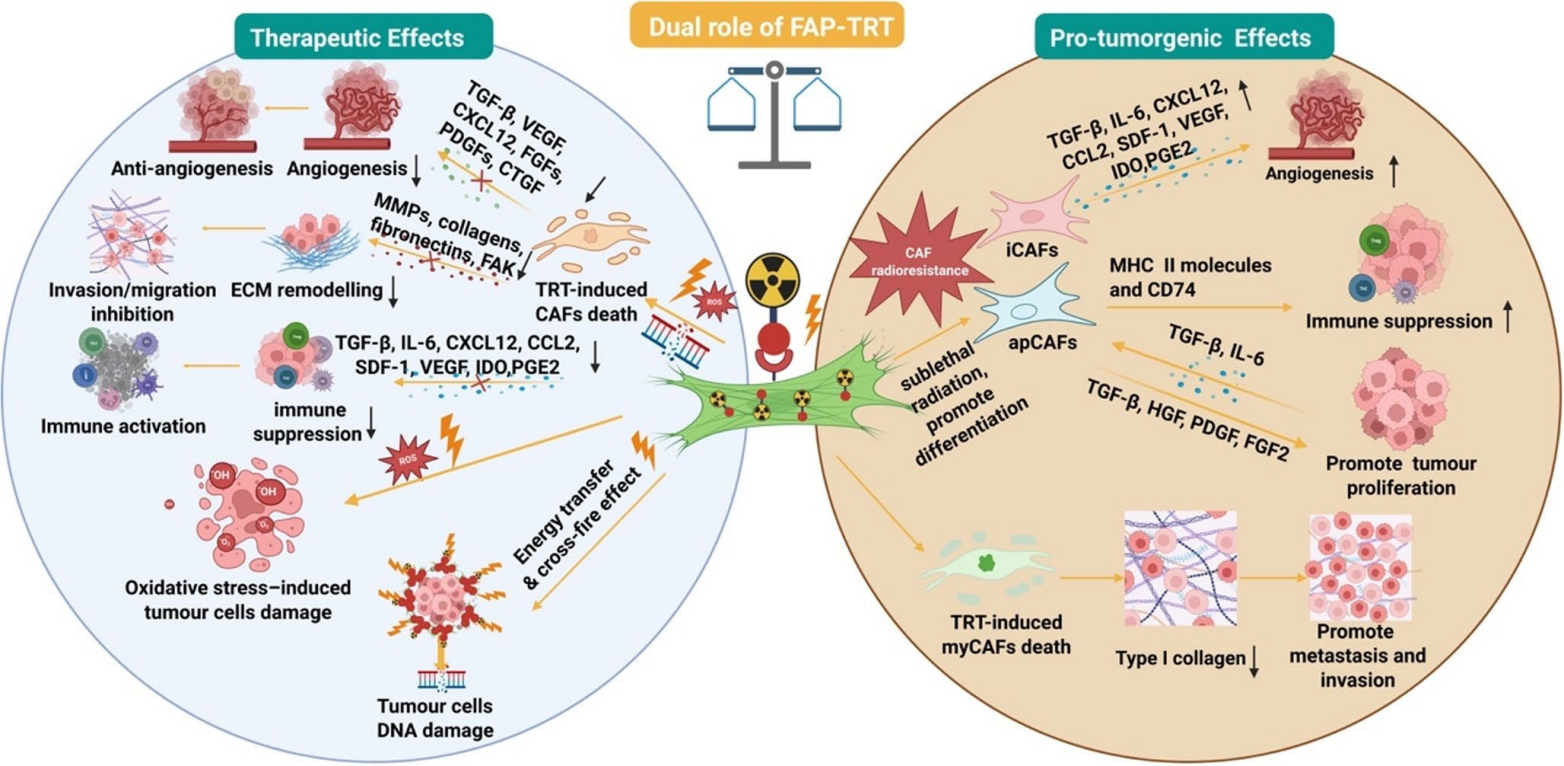
Schematic illustration of the dual roles of fibroblast activation protein (FAP)-targeted radionuclide therapy (TRT) on the tumour microenvironment (TME), highlighting both therapeutic and pro-tumorigenic effects. Abbreviations: CAFs, cancer-associated fibroblasts; iCAFs, inflammatory CAFs; apCAFs, antigen-presenting CAFs; myCAFs, myofibroblastic CAFs; TGF-β, transforming growth factor beta; IL-6, Interleukin-6; VEGF, vascular endothelial growth factor; CXCL12, C-X-C motif chemokine ligand 12; CCL2, C-C motif chemokine ligand 2; SDF-1, stromal cell-derived factor 1; IDO, indoleamine 2,3-dioxygenase; PGE2, prostaglandin E2; MMPs, matrix metalloproteinases; FAK, focal adhesion kinase; ROS, reactive oxygen species. (Created in BioRender. Wu, C. (2025) https://BioRender.com/gyb5y68)

Several single-cell transcriptomic studies have further revealed that FAP-positive (FAP⁺) CAFs are not a uniform population but comprise multiple transcriptionally and functionally distinct subtypes [83, 84]. Three CAF subtypes are reported, including immune-regulatory CAFs (iCAFs), tumour-restraining myofibroblastic CAFs (myCAFs) and antigen-presenting CAF (apCAF) [85], exhibiting distinct functional subtypes that can either promote or suppress tumour progression (Fig. 2) [86–88]. Notably, FAP⁺ CAFs commonly consist of inflammatory (iCAF) and myofibroblastic (myCAF) subclasses. iCAFs are typically enriched near tumour margins and secrete cytokines such as IL-6, whereas myCAFs localise closer to cancer cells and display high extracellular matrix (ECM)-remodelling activity [84]. Furthermore, myCAFs could play roles in suppressing tumour proliferation and enhancing immune surveillance, and perturbation of myofibroblastic components in the pancreatic ductal adenocarcinoma (PDAC) TME might promote tumour progression [87]. While FAP-TRT aims to eradicate FAP-expressing CAFs to disrupt tumour-stromal interactions, it is crucial to recognise that not all CAF subtypes contribute to tumour growth. Moreover, the composition of CAF subtypes not only varies across tumour types but also displays significant inter-patient heterogeneity [84]. iCAFs and myCAFs have been consistently identified across several tumour entities, including breast cancer [89], pancreatic ductal adenocarcinoma [90], cholangiocarcinoma [91], head and neck squamous cell carcinoma [84], and non-small cell lung cancer [84]. These inter-tumour and inter-patient differences in CAF subtype prevalence have important implications for FAP-TRT efficacy, as targeting all FAP^+^ CAFs indiscriminately may remove tumour-restraining CAF subsets in certain contexts while being therapeutically advantageous in others.

Accumulating evidence from external beam radiotherapy indicates that fibroblasts in the tumour stroma frequently undergo radiation-induced premature senescence rather than apoptosis, particularly under sublethal doses [92]. Such radiation-induced senescent CAFs acquire a stable pro-tumorigenic phenotype characterised by increased secretion of cytokines and growth factors, including transforming growth factor beta (TGF-β), C-X-C motif chemokine ligand 12 (CXCL12), Interleukin-6 (IL-6), C-C motif chemokine ligand 2 (CCL2), stromal cell-derived factor 1 (SDF-1), vascular endothelial growth factor (VEGF), indoleamine 2,3-dioxygenase (IDO), prostaglandin E2 (PGE2), HGF and matrix-remodelling enzymes such as matrix metalloproteinase (MMPs) [92–94]. These secreted molecules can enhance angiogenesis, suppress the immune system, promote proliferation, and migration.

Taken together, these findings establish that radiation can reprogram CAFs toward a more tumour-supportive state. Given that TRT also delivers localised radiation to the TME, it is plausible that FAP-TRT may similarly induce therapy-activated CAFs subsets when radiation exposure is heterogeneous or sublethal. This duality highlights that while FAP-TRT aims to deplete FAP + CAFs, incomplete CAFs eradication may instead promote pro-tumorigenic CAFs reprogramming, underscoring the importance of considering CAFs heterogeneity and radiation sensitivity when optimising FAP-TRT strategies.

### Impact and uncertainty of bystander and immune-mediated effects

One of the unique aspects of FAP-TRT is its potential to induce not only direct cytotoxicity in FAP-expressing stromal cells but also antitumour effects, such as bystander effects and abscopal effects [95]. These refer to radiation-induced damage in neighbouring or even distant tumour cells that do not exposed to radiation. Although bystander and abscopal effects are generally regarded as beneficial in enhancing the efficacy of TRT, these mechanisms may paradoxically contribute to tumour progression under certain conditions. Potential risks include inflammation-induced proliferation, pro-tumourigenic signalling from damaged CAFs via TGF-β, HGF, PDGF, FGF2 pathways, immune exhaustion following transient activation, and ECM remodelling that facilitates invasion and metastasis [96, 97].

Moreover, TRT generates reactive oxygen species (ROS) through water radiolysis, contributing to its cytotoxic effects [98]. However, ROS are also known to promote fibroblast activation and CAF differentiation [96]. Therefore, under sublethal radiation exposure, CAFs may not be eliminated but instead become further activated, potentially enhancing their pro-tumourigenic functions [99]. This phenomenon underscores the need for careful optimisation of radionuclide dose selection, ensuring that sufficient radiation is delivered to CAFs to achieve a therapeutic effect while minimising unintended consequences.

Importantly, such effects carry significant implications for clinical translation. Preclinical mouse models, particularly immunodeficient or subcutaneous tumour models, often fail to recapitulate the intricate tumour–immune–stroma dynamics found in patients. While these models have been instrumental in mechanistic studies, their predictive value for human therapy is limited. TME is composed not only of tumour cells but also includes various other components such as immune cells, stroma, blood vessels, and adipocytes [99]. The immune system in murine models, particularly in immunodeficient or subcutaneously implanted tumour models, does not accurately mimic the intricate immune-tumour-stroma interactions present in human patients. Studies suggested that CAFs can recruit immune cells and transform them into immunosuppressive phenotypes regarding tumour progression [100–102]. As a result, preclinical data may overestimate or underestimate the clinical relevance of these effects, limiting the predictive value of animal studies in guiding human therapy. This gap underscores the need for improved translational models and more comprehensive clinical investigation to clarify the therapeutic contributions and limitations of bystander and abscopal mechanisms in FAP-TRT.

### Impact of tumour size on therapeutic efficacy

Studies have shown that tumour size can markedly affect the efficacy of targeted radionuclide therapy [103, 104]. In preclinical models, smaller tumours (< 1 cm^3^) responded more effectively to radiolabelled agents such as ¹¹¹In- or ¹⁷⁷Lu-labelled somatostatin analogues, whereas larger tumours exhibited reduced therapeutic response [103, 104]. Building on these findings, tumour size also plays a critical role in determining the effectiveness of FAP-TRT, particularly in studies evaluating different radiolabelled FAP ligands (e.g., ²²⁵Ac, ¹⁶¹Tb, ¹⁷⁷Lu) [105]. The efficacy of these agents depends not only on their intrinsic properties, but also on their ability to penetrate the tumour and bind to FAP-expressing CAFs. In larger tumours, limited vascular perfusion and increased stromal heterogeneity may impair radiopharmaceutical delivery and FAP accessibility, thereby reducing therapeutic uptake and compromising efficacy. Therefore, applying FAP-TRT across tumours of varying sizes may provide valuable insights into how tumour size influences target engagement and therapeutic response. Such investigations are essential for optimising the clinical translation of FAP-TRT and understanding the spatial limitations of its targeting efficiency.

## Future directions and strategies for improvement

### Enhancing translation

To strengthen the translational value of preclinical studies, it is crucial to use models that better replicate the complexity of human tumours, especially the interaction between CAFs, immune cells, and tumour cells. While conventional subcutaneous xenografts in immunodeficient mice remain widely used, they often fail to capture this complexity.

Future preclinical studies should prioritise models that directly address these limitations. First, orthotopic models that allow tumours to grow in the native tissue context should be adopted, as they preserve organ-specific microenvironment and immune interactions, thereby enhancing clinical relevance. Second, syngeneic mouse models with intact immune systems should be used to enable the assessment of immune-mediated and stromal responses to FAP-TRT. Third, when using PDX, lower passage numbers should be preferred to retain tumour-stroma architecture and heterogeneity, ensuring predictive accuracy. Finally, chimeric mouse models incorporating human CAFs or humanised stroma offer a promising approach to study human-specific stromal targeting effects of FAP-TRT. These strategies provide concrete recommendations to overcome current limitations and improve the accuracy of preclinical findings, thereby guiding more effective clinical translation. Moreover, emerging advanced preclinical models such as coculture patient-derived organoids and organoid-on-chip systems represent promising tools to better recapitulate the complex tumour-stroma-immune interactions in vitro, offering valuable platforms for translational research in FAP-TRT.

### Personalised dosimetry and patient selection

Several clinical studies have reported substantial inter- and intra-patient variability in tumour-absorbed doses of FAP-TRT [16, 19, 26]. Given the inter- and intra-patient heterogeneity in FAP expression and tumour perfusion, pre-therapeutic dynamic PET-derived kinetic metrics such as Ki, K1, and the time–activity curve (TAC) slope may provide quantitative insights into tracer uptake and clearance, thereby allowing more accurate prediction of tumour-absorbed doses and individualised dosimetry in FAP-TRT. As discussed above, suboptimal radiation delivery may not only fail to deplete CAFs but may paradoxically trigger pro-tumourigenic pathways through sublethal activation. Therefore, personalised dosimetry has emerged as a crucial strategy, allowing assessment of whether the tumour is likely to receive an effective therapeutic dose without exceeding the tolerance of healthy organs.

In parallel, tissue-based markers such as FAP immunohistochemistry, stromal density, immune infiltration profiles, or genetic alterations related to CAF activation and DNA damage repair may serve as valuable tools for selecting patients most likely to benefit from FAP-TRT.

### Combination approaches

To overcome the limitations of monotherapy, combination strategies are emerging as a promising direction. FAP-TRT may synergise with conventional chemotherapy or external beam radiotherapy, exploiting the tumour stroma’s role in drug delivery and radiation response modulation [106]. Moreover, coupling FAP-TRT with immunotherapy holds particular potential, as localised radiation can induce immunogenic cell death and enhance tumour antigen presentation, thereby priming the immune system for checkpoint inhibition or adoptive cell therapies. These combination approaches may ultimately amplify therapeutic efficacy and broaden the clinical applicability of FAP-TRT.

However, the true therapeutic contribution of FAP-TRT in these combinations remains to be established. It is essential to first demonstrate the efficacy of FAP-TRT as a monotherapy. Future randomised controlled trials, such as those comparing chemotherapy alone versus chemotherapy plus FAP-TRT, are necessary to rigorously assess the additive or synergistic benefit of FAP-TRT and to guide clinical implementation.

## Conclusion

The clinical translation of FAP-TRT faces major challenges, including the selection of appropriate tumour models, tumour size–dependent uptake, and heterogeneity in FAP expression. Refining preclinical models to better replicate human tumour-stroma-immune interactions remains essential. In clinical settings, personalised dosimetry is critical to balance therapeutic efficacy with radiobiological safety. Patient stratification using imaging or molecular biomarkers may further improve target engagement and outcome prediction. Finally, rational combination strategies—such as pairing FAP-TRT with immunotherapy—may enhance precision and maximise clinical benefit.

## Data Availability

Data sharing not applicable to this article as no data-sets were generated or analysed during the current study.
